# The Roles of Traditional Chinese Medicine: Shen-Fu Injection on the Postresuscitation Care Bundle

**DOI:** 10.1155/2013/319092

**Published:** 2013-08-28

**Authors:** Qian Zhang, Chunsheng Li

**Affiliations:** Department of Emergency Medicine, Beijing Chaoyang Hospital, Capital Medical University, 8 Workers' Stadium South Road, Chaoyang District, Beijing 100020, China

## Abstract

Survival rates following in-hospital and out-of-hospital cardiac arrests remain disappointingly low. Organ injury caused by ischemia and hypoxia during prolonged cardiac arrest is compounded by reperfusion injury that occurs when a spontaneous circulation is restored. A bundle of procedures, which may need to be administered simultaneously, is required. The procedures include prompt identification and treatment of the cause of cardiac arrest, as well as a definitive airway and ventilation together. Additional benefit is possible with appropriate forms of early goal-directed therapy and achieving therapeutic hypothermia within the first few hours, followed by gradual rewarming and ensuring glycaemic control to be within a range of 6 to 10 mmol/L. All these would be important and need to be continued for at least 24 hours. Previous studies have showed that the effects of Shen-Fu injection (SFI) are based on aconitine properties, supplemented by ginsenoside, which can scavenge free radicals, improve energy metabolism, inhibit inflammatory mediators, suppress cell apoptosis, and alleviate mitochondrial damage. SFI, like many other complex prescriptions of traditional Chinese medicine, was also found to be more effective than any of its ingredient used separately in vivo. As the postresuscitation care bundle is known to be, the present paper focuses on the role of SFI played on the postresuscitation care bundle.

## 1. Introduction

Cardiac arrest (CA) results in whole-body ischemia/reperfusion and represents the most severe shock state, during which the delivery of oxygen and metabolic substrates is abruptly halted, and metabolites are no longer removed. Cardiopulmonary resuscitation (CPR) only partially reverses this process [1]. Furthermore, the myocardium is a main target tissue during this form of ischemia/reperfusion, and the destruction of tissue progresses even after circulation has been successfully restored. 

In patients who initially achieve return of spontaneous circulation (ROSC) after CA, the significant subsequent morbidity and mortality are largely due to the cerebral and cardiac dysfunction that accompanies prolonged whole-body ischemia. CA contributes to hemodynamic disorders that cause the systemic release of massive oxygen free radical, lactic acid, and metabolites of arachidonic acid, which could reach the different tissues by blood circulation and cause ischemia/reperfusion injury [2, 3]. Inadequate tissue oxygen delivery can persist even after ROSC; accumulated oxygen debt leads to activation of immunologic pathways and systemic inflammation, which increases the risk of multiple organ failure. This condition had many features in common with sepsis [4]. The pathophysiological changes cause multiple organ dysfunctions and have recently been termed the “postcardiac arrest syndrome,” which comprises anoxic brain injury, postcardiac arrest myocardial dysfunction, systemic ischemia/reperfusion response, and persistent precipitating pathology [5]. 

**Figure 1:**
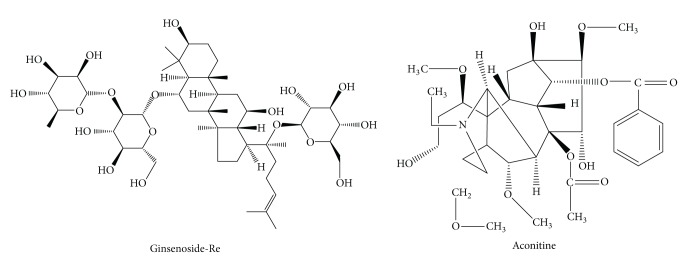
The chemical structures of ginsenoside-Re and Aconitine.

Shen-Fu injection (SFI), which has been commonly used in China for nearly 800 years, is a well-known traditional Chinese medical formulation containing ginseng (*Panax*; family: Araliaceae) and fuzi (Radix aconiti lateralis preparata, *Aconitum carmichaeli* Debx.; family: Ranunculaceae), which is produced using multistage countercurrent extraction and macroporous resin adsorption technology (Ya'an Sanjiu Pharmaceutical Co., Ltd., China). Its quality is controlled strictly according to the standard of China Ministry of Public Health and fingerprint technology was adopted in the process of production to ensure its quality [6]. Their chemical structures are as shown in Figure 1.

Lots of clinical and epidemiological studies have demonstrated that SFI has significant protective effects on ischemia/reperfusion injury of the brain, spinal cord, kidney, intestine, liver, and especially the heart [7–11]. However, relatively little is known about the effects of SFI on postresuscitation care bundle. The present paper focuses on the association between postresuscitation care bundle and SFI and explains the roles of SFI on the postresuscitation care bundle treatment.

## 2. Postresuscitation Care Bundles

Bundles are a group of “therapies” built around the best evidence-based guidelines, which, when implemented together, produce greater benefit in terms of outcome than the individual therapeutic interventions [12]. The conception of care bundles has been proposed based on the holistic principle that the whole is greater than the sum of its parts. Bundles play a useful role to help remove the constraints imposed by these deviations and variations by means of constructing the elements into packages that must be implemented in strict compliance for every patient, at each and every single time to ensure uniformity as well as universality [13]. 

In the year of 2005, the final ring of the chain of survival was updated to reflect the importance of postresuscitation care in determining the ultimate outcome following CA [14]. Organ injury caused by ischemia and hypoxia during prolonged CA is compounded by reperfusion injury that occurs when a spontaneous circulation is restored. These insults trigger a systemic inflammatory response, similar to that associated with sepsis [15]. Here, we propose a “postresuscitation care bundle” which comprises (1) early coronary reperfusion and hemodynamic optimization, (2) airway and ventilation management, (3) therapeutic hypothermia, (4) neurological enhancement measures and monitoring, and (5) glycaemic control [16].

### 2.1. Early Coronary Reperfusion and Hemodynamic Optimization

Patients resuscitated from CA who have electrocardiographic criteria for ST-elevation myocardial infarction should undergo immediate coronary angiography, with subsequent percutaneous coronary intervention (PCI) if indicated. If PCI is not available, thrombolytic therapy is an appropriate alternative for postcardiac arrest management of ST-elevation myocardial infarction [17]. This may also be helpful for monitoring blood pressures during therapeutic hypothermia and for accurate measurement of hemodynamic parameters so as to determine the most appropriate combination of medications for maintenance of perfusion. Central venous pressure monitoring would also be useful. 

### 2.2. Airway and Ventilation Management

Airway control is crucial in the initial stages of postresuscitation management. Insertion of a definitive airway (if not done yet) guided by capnography is followed by a chest radiograph for confirmation of tube position. While mechanical ventilation would be required so as to reduce the work of breathing, the rate of ventilation and tidal volume would need to be adjusted in order to maintain arterial oxyhemoglobin saturation at ≥94% [18, 19]. The concerns that hyperoxaemia during the reperfusion phase after ROSC with 100% oxygen may lead to increased brain lipidperoxidation, increased metabolic dysfunction, and neurological degeneration, as well as concerns about its impact on short-term functional outcome, have resulted in calls of ventilation with room air or an inspired oxygen fraction titrated to maintain a pulse oximetry reading of 94% to 98% [20]. Weaning the patients from 100% oxygen to the FiO_2_ required to maintain SpO_2_ at the above stated levels should begin once ROSC is achieved [21].

### 2.3. Therapeutic Hypothermia

It has been well demonstrated that brain temperatures during the first 24 hours after ROSC have a significant effect on survival and neurological recovery in patients who remain comatose soon after ROSC [22]. Preclinical and clinical evidence strongly supports mild therapeutic hypothermia as an effective therapy for the postcardiac arrest syndrome [23]. Unconscious adult patients with spontaneous circulation after out-of-hospital VF cardiac arrest should be cooled to 32–34°C for at least 12 to 24 hours. Induced hypothermia might also benefit unconscious adult patients with spontaneous circulation after out-of-hospital cardiac arrest from a nonshockable rhythm or in-hospital cardiac arrest [24]. 

### 2.4. Neurological Enhancement Measures and Monitoring

Postcardiac arrest brain injury is a result of initial ischemic injury followed by reperfusion injury occurring within the hours and days after ROSC [25]. Features indicating occurrence of brain injury in post-ROSC patients include coma, seizures, myoclonus, and various degrees of neurocognitive dysfunction, ranging from memory deficits to a persistent vegetative state and finally brain death. The neurological prognosis in the majority of comatose CA survivors cannot be reliably predicted until at least 72 hours after CPR. Currently, the principal neuroprotective measures recommended include normoventilation with controlled oxygenation to avoid hyperoxaemia and minimize the likelihood of lowering cerebral perfusion or aggravating cerebral ischemia, achieving normoglycaemia to optimize neuronal recovery and therapeutic hypothermia to minimize the cerebral and multisystem metabolic functions until biochemical and cellular parameters are better optimized [26].

### 2.5. Glycaemic Control

Hyperglycaemia occurring post-ROSC has been associated with increased mortality and worse neurological outcomes [27]. Similarly, hypoglycaemia is also associated with poor outcomes in critically ill patients [28]. The strategy is to maintain blood sugar levels at 6–10 mmol/L. Blood glucose concentrations must be monitored frequently in these patients, and hyperglycemia should be treated with an insulin infusion. A target glucose range with an upper value of 8.0 mmol/L (144 mg/dL) has been suggested by others [29–31]. The lower value of 6.1 mmol/L (110 mg/dL) may not reduce mortality any further but instead may expose patients to the potentially harmful effects of hypoglycemia [29]. The incidence of hypoglycemia in another recent study of intensive insulin therapy exceeded 18%, and some have cautioned against its routine use in the critically ill patients [32–34]. Regardless of the chosen glucose target range, blood glucose must be measured frequently, especially when insulin is started and during cooling and rewarming periods.

## 3. The Roles of SFI on the Postresuscitation Care Bundle Treatment

### 3.1. SFI Mitigates the Postresuscitation Myocardial Dysfunction

Postresuscitation myocardial dysfunction has been implicated as one of the major causes of fatal outcomes in patients who fail to survive hospitalization after CPR [35]. Results from Ji et al. showed that SFI significantly increased mean arterial pressure and improved the cardiac output and ejection fraction after ROSC. SFI also can attenuate postresuscitation myocardial dysfunction through beneficial effects on energy metabolism and remarkable antioxidant capacity. Meanwhile, it is also demonstrated that SFI can prevent and cure different kinds of arrhythmia (tachycardia and bradycardia), while such two-way regulating effect has rarely been found in western medicine [36]. Furthermore, in an in vitro study, Gu et al. reported the cardioprotective effects of SFI by decreasing myocardial injury, improving myocardial ultrastructure, inhibiting Bcl-2, Bax, and caspase 3 expressions, and reducing myocardial apoptosis [37]. 

### 3.2. SFI Extenuates Postresuscitation Lung Injury

Since post-ROSC patients are at risk of acute lung injury or acute respiratory distress syndrome, the standard recommendation for ventilation would be normocapnia. Excessive tidal volumes would not also be recommended owing to the risks of increased intrathoracic pressures with attendant reduced venous return and cardiac output. Zhang et al. have reported that SFI resulted in improving oxygenation index, respiratory index, oxygen extraction ratio, dynamic lung compliance, airway resistance, external vascular lung water index, and pulmonary vascular permeability index, which indicates that SFI can effectively protect pulmonary gas exchange function, increase oxygen consumption and extraction, decrease structural lung damage, and alleviate pulmonary edema after ROSC [38]. A recent study has also demonstrated that SFI could reduce postresuscitation lung injury and improve lung immune function by regulating lung imbalance of Th1/Th2 [39].

### 3.3. SFI Alleviates Postresuscitation Cerebral Injury

In an in vitro study, Yang et al. showed that cerebral injury is aggravated progressively at the early phase following CPR. SFI could alleviate cerebral injury after CPR, and this protective effect might be of dosage effect relationship. Large dosage of SFI could promote the expression of neuron-specific enolase in neurons,which might alleviate the cerebral injury after CPR [40].

### 3.4. SFI and Glycaemic Control

Hyperglycemia is common after CA due to the upregulated stress response. Tight blood glucose control remains controversial in the critical care setting. It has been shown to improve survival in a surgical intensive care setting, but this outcome did not convey to the medical intensive care setting [41]. The blood glucose concentration should be monitored frequently in the postcardiac arrest syndrome patient, especially when instituting the therapeutics hypothermia. In an in vitro study, Shan-shan et al. reported that SFI could increase insulin secretion by increasing PI3K expression, which can improve hyperglycemia during CPR. The effects were likely caused by increased PI3K contents in islets. The mechanism may be partly related to SFI reversing the disequilibrium of oxidization and antioxidation [42]. 

## 4. The Holistic Approach with SFI

### 4.1. The Effect of SFI on Na^+^-K^+^-ATPase/Ca^2+^-ATPase Activity

Na^+^-K^+^-ATPase activity is an electrogenic process in which two Na^+^ ions extrude out of the cell, whereas one K^+^ ion enters the cell, thereby maintaining an appropriate transmembrane Na^+^ gradient. Ca^2+^-ATPase enzyme is another sarcolemmal enzyme. Intracellular calcium loading is considered to represent the common denominator of ischemia/reperfusion-induced cell dysfunction and death. Various studies showed that Na^+^-K^+^-ATPase and Ca^2+^-ATPase enzymes may play a key role in the prevention of ischemia/reperfusion [43–45]. Luo et al. have found that SFI could restore the ability of Na^+^-K^+^-ATPase and Ca^2+^-ATPase enzyme activities, and it may be one of the mechanisms by which SFI could attenuate the postresuscitation myocardial dysfunction [46]. Additionally, the effect of SFI's blockage on the sodium channels in cardiac myocytes may be one of the important molecular mechanisms for its cardiac active effectiveness.

### 4.2. Effects of SFI on Oxygen Free Radicals

CPR can be viewed as a process of whole-body ischemia/reperfusion [47]. A large amount of oxygen free radicals (OFRs) produced during the ischemia/reperfusion play a major role in myocardial damage. Malondialdehyde (MDA) is an end product of lipid peroxidation that causes cellular damage and disruption of cell membranes when tissue antioxidants are exhausted. MDA has been found to increase in myocardial tissue after myocardial ischemia/reperfusion [48]. Superoxide dismutase (SOD) is a metalloenzyme that catalyzes the dismutation of O_2_^−^ into O_2_ and H_2_O_2_ and affords protection against OFRs damage [49]. The activity of SOD could reflect the in vivo scavenging capability of OFRs. Reducing of MDA content and attenuating the decrease of SOD activities in myocardial tissue have cardioprotective effects [50]. A recent study has shown that, comparing with saline group, A recent study has shown that the MDA content was significantly decreased in SFI-treated myocardium subjected to ischemia/reperfusion injury. At the same time, the activities of serum SOD in the SFI group were significantly higher than in the saline group [51]. These results indicate that SFI can reduce MDA content, attenuate the decrease of SOD activities in myocardial tissue, and thus alleviate the degree of myocardial injury. 

### 4.3. SFI Mitigates the Impact of Calcium Overload

Ca^2+^ is a ubiquitous signal for regulating cellular function, including survival and death [52]. While a small amount of Ca^2+^ is necessary for the optimal physiological function of the heart, growing evidence suggests that an increased cytosolic free Ca^2+^ overload is one of the major contributors of myocardial injury induced by ischemia/reperfusion [53–55]. Therefore, Ca^2+^ handling in the postischemic myocardium has become a prime target. A recent study showed that the protective effects of SFI on myocardial ischemia/reperfusion injury were realized by repressing the opening of Ca^2+^ channel myocardial cell membrane, reducing inflow of Ca^2+^, inhibiting calcium overload, decreasing OFRs, and suppressing inflammation [56]. These results indicate that SFI can mitigate the impact of calcium overload.

### 4.4. The Relationship between SFI and PI3K/Akt Signaling Pathway

Some researches have showed that PI3K/Akt signaling pathway plays a crucial role in protecting the myocardium from ischemia/reperfusion injury [57, 58]. Strong evidence shows that endothelial nitric oxide synthase (eNOS) is an important target of Akt and eNOS plays the role of an important cardiovascular protective molecule [59]. Recent studies show that increased expression of Akt and eNOS alleviates the ischemia/reperfusion injury [60, 61]. Wu et al. showed that SFI treatment resulted in an enhanced level eNOS which was significantly higher than that of saline-treated ischemia/reperfusion group [62]. These results suggested that SFI-induced cardioprotective effects are mediated through Akt-induced eNOS phosphorylation. 

### 4.5. SFI Prevents Cardiomyocytes Apoptosis

In a present study, after 24 hours post-ROSC, significant myocardial damage and apoptosis emerged, accompanied by increased protein expression of Bcl-2, Bax, and active caspase-3 [63]. The most principal pathways for apoptosis initiation are termed the Bcl-2/Bax-controlled pathway. Furthermore, downregulation of Bcl-2/Bax expression after postresuscitation might result in the activation of the caspase family of proteases, such as caspase 3, which is responsible for the induction of apoptotic cell death, leading to internucleosomal DNA fragmentation [64]. In an in vitro study, Wang et al. reported that treatment with different doses of SFI protected cardiac myocyte cultures from hypoxia/reoxygenation-induced apoptosis. Caspase-3 activation was decreased in hypoxic/reoxygenated cardiomyocytes cotreated with SFI when compared to hypoxia/reoxygenation alone treated cultures. Expression of the Bcl-2 proteins was increased in SFI-treated cardiomyocytes subjected to hypoxia/reoxygenation [65]. These results showed that SFI could significantly attenuate postresuscitation myocardial dysfunction by modulating apoptosis.

## 5. Conclusion

In summary, postcardiac arrest syndrome is associated with complex pathophysiological changes and a high mortality rate, and a multidisciplinary approach to treatment is optimal. SFI might be useful in the treatment of postcardiac arrest syndrome, incorporating the multilayer and multitarget advantages of TCM. Above all, SFI plays a key role during postresuscitation care bundle. Furthermore, with the initiation of large-scale, multicenter studies and further research into the pharmacological actions of SFI, we believe that SFI will eventually be widely used for postresuscitation care bundle treatment.
